# Teamwork and safety climate in Polish long-term care facilities: questionnaire reliability and usability

**DOI:** 10.1038/s41598-023-48415-8

**Published:** 2023-11-30

**Authors:** Jakub Świtalski, Agnieszka Radomska, Tomasz Tatara, Katarzyna Wnuk, Wojciech Miazga, Rafał Szpakowski, Mariola Borowska, Agnieszka Zdęba-Mozoła, Remigiusz Kozłowski, Michał Marczak, Aleksandra Czerw, Grażyna Dykowska

**Affiliations:** 1 Department of Health Economics and Medical Law, Faculty of Health Sciences, Medical University of Warsaw, ul. Erazma Ciołka 27, 01-445 Warszawa, Poland; 2 Department of Public Health, Faculty of Health Sciences, Medical University of Warsaw, 02-091 Warsaw, Poland; 3 Department of Health Policy Programs, Faculty of Health Technology Assessment, Agency for Health Technology Assessment and Tariff System, 00-032 Warsaw, Poland; 4 School of Public Health, Centre of Postgraduate Medical Education of Warsaw, 01-826 Warsaw, Poland; 5 Department of Development of Nursing, Social and Medical Sciences, Faculty of Health Sciences, Medical University of Warsaw, 01-445 Warsaw, Poland; 6 Department of Cancer Epidemiology and Prevention, Maria Sklodowska-Curie National Research Institute of Oncology, 02-781 Warsaw, Poland; 7 Department of Management and Logistics in Healthcare, Medical University of Lodz, 90-131, Lodz, Poland; 8 Center for Security Technologies in Logistics, Faculty of Management, University of Lodz, 90-237 Lodz, Poland; 9 Collegium Management, WSB Merito University in Warsaw, 03-204 Warszawa, Poland; 10 Department of Economic and System Analyses, National Institute of Public Health NIH-National Research Institute, 00-791 Warsaw, Poland; 11 Warsaw College of Engineering and Health, 02-366 Warsaw, Poland

**Keywords:** Health care, Geriatrics, Health services, Public health

## Abstract

The Teamwork and Safety Climate Survey (TSCS) is one of the questionnaires used to measure patient safety. The questionnaire includes two scales: teamwork climate and safety climate. The objective of the study is the linguistic and cultural adaptation of the TSCS to Polish conditions and checking the reliability and usability of the tool in long-term care facilities. Firstly, the TSCS was translated into Polish. Then, a cross-sectional survey was conducted among the medical and auxiliary personnel of long-term care facilities all over Poland. The psychometric properties of the questionnaire were analysed (including Cronbach’s alpha coefficient). Correlations between the areas of the questionnaire and individual variables relating to facility parameters were also calculated. Respondents (n = 558) working in 26 different long-term care facilities participated in the study. The analysis has provided four scales instead of two of the original version of the questionnaire (teamwork climate, safety climate, ability to speak up and following the rules, work organisation). Correlation analysis revealed a number of significant correlations between the scales and individual variables corresponding to the parameters of long-term care facilities and respondents themselves. In conclusion, the Polish version of the TSCS may be a useful tool to measure aspects related to patient safety culture in long-term care facilities.

## Introduction

Ensuring patient safety is one of the most important elements of the functioning healthcare system. In recent years, steps have been taken to ensure the appropriate quality and safety of care^1–4^. In practice, actions are taken to increase the culture of safety, which can be defined as the result of individual and group values, attitudes, competences, and behavioral patterns that determine the level of involvement in the organization of care and its safety^1,5–7^. This is particularly important in long-term care facilities where patients with multiple diseases stay, especially in times of increasing demand for this type of care and shortages of medical personnel observed in Poland. Here it is necessary to follow the rules of conduct in accordance with the latest medical knowledge, minimizing the risk of adverse events^8,9^.

Medical personnel play a key role in patient care. Therefore, a comprehensive assessment of the safety culture of healthcare facilities should involve a study of the impact of the human factor on patient safety^10^. Due to the nature of the tasks performed, the work of nurses, medical caregivers, doctors, physiotherapists and other people dealing with patients is subject to a high risk of error. Therefore, it is necessary to strive to optimize the procedures and behaviour of medical personnel, so that the number of adverse events and errors is reduced to a minimum. The cooperation of therapeutic teams and the appropriate communication within them can contribute to improving the patients’ situation^11,12^. An important role in the process of creating a safe climate is also played by facility management^13^.

In Poland, the implementation of formal long-term care is based on both public (health and social services) and private sectors. In the public sector of health services, it is possible to take advantage of residential care provided in long-term care facilities.

A referral to a long-term care facility is granted to a person who requires 24-h nursing and care services, rehabilitation and the continuation of treatment, and does not require hospitalization in a hospital ward, and who has obtained 40 points or less on the scale of the level of dependence (Barthel scale). Individuals with a Barthel score of 40 or less are not admitted to these facilities, if the main indication for care is advanced cancer, mental illness or addiction. The stay in the facility is payable. The fee is determined by the manager of a given facility, based on documents confirming the patient’s income. The person admitted to the facility bears the costs of accommodation and meals. The monthly payment is set at 250% of the lowest old-age pension. However, the fee cannot be more than 70% of the patient’s monthly income. Health services are financed from the budget of the public payer, which in Poland constitutes the National Health Fund.

Over the past dozen or so years, there has been a significant increase in the number of long-term care facilities. In 2021, the number of long-term care facilities with 29,282 beds was 527. Compared to 2000, the number of facilities increased by 352, and the number of beds by 17,849^14^. Despite significant development, the demand for residential long-term care services still significantly exceeds supply.

There are numerous tools to measure patient safety culture. These include Surveys on Patient Safety Culture (SOPSC), The Safety Attitudes Questionnaire (SAQ), The Manchester Patient Safety Framework (MaPSaF), and The Patient Safety Climate in Healthcare Organizations (PSCHO)^15,16^.

The Teamwork and Safety Climate Survey (TSCS) is one of the questionnaires used to measure patient safety. It contains statements that have been used in various SAQ versions^17–20^. The questionnaire includes two scales: the teamwork climate (statements 1–14) and the safety climate (statements 15–27)^21^.

The number of studies on patient safety culture in long-term care facilities conducted in the world is small, and a significant part of them was conducted in the USA, using the Nursing Home Survey on Patient Safety Culture (NHSOPSC)^22,23^. Several publications that used the SAQ in long-term care facilities were also identified^24–28^.

The objective of the study is the linguistic and cultural adaptation of the TSCS to Polish conditions and checking the reliability and usability of the tool in long-term care facilities.

## Methods

### Translation procedures and cultural adaptation

The TSCS was translated into Polish after prior approval from the Center for Healthcare Quality and Safety, Science Center at Houston, The University of Texas Health. The cultural adaptation was done in accordance with good practices in this field (investigation of conceptual and item equivalence, translation, synthesis, back translation, expert committee review, pretesting, main study)^29^.

The questionnaire was translated into Polish by two independent translators familiar with patient safety issues. The translators also prepared a reports in which they included their comments and doubts regarding some of the phrases. Two translated versions and reports were analyzed, and a synthesized version of the translation was constructed. All discrepancies were resolved by consensus. A third translator then back translated this questionnaire version to ensure that the translation was done correctly. Then, the questionnaire in English and Polish was presented to experts in the field of patient safety, who did not raise any objections.

In the last stage of translation process, 20 people (doctors, nurses and medical caregivers) were asked to read the questionnaire and complete it. Then they were asked if all the statements were clear and unambiguous. In addition, questions were asked about potential changes to the questionnaire that could be beneficial for its comprehensibility and reception. No substantive comments were submitted regarding the questions in the questionnaire. Simultaneously, respondents presented several technical comments regarding the font used and content layout in the part with additional questions, concerning professional position and experience in organization. After taking them into account, the final Polish version of the questionnaire was prepared.

The questionnaire, in terms of response categories, remained unchanged (a translated into Polish 5-point Likert scale). In our opinion, the scale used in the questionnaire is optimal and allows for comparison of results between versions translated into other languages and its original version. A graphic form similar to the original version of the questionnaire was used.

### Scoring

The TSCS consists of 27 statements (Supplementary Information). The tool uses a 5-point Likert scale [strongly disagree (1 point on the scale)—rather agree (2 points on the scale)—neither agree nor disagree (3 points on the scale) —rather agree (4 points on the scale)—I strongly agree (5 points on the scale)]. In the absence of adequacy of the statement asked in relation to the characteristics of the respondent, it is possible to check the box “this statement does not apply to me”. At the time of selecting the option, the statement is not included in the overall score of a given respondent.

In accordance with the adopted methodology, to calculate the value of a specific scale, the average of all statements from the scale that the respondent answered should be calculated (the score for individual answers is presented above). In the case of some items, the scoring is reversed, i.e., for the answer “I strongly disagree” the respondent receives 5 points, and for the answer “I strongly agree”—1 point.

The formula for calculating—the score of a single respondent: (((the mean for statements on a scale with inverse scoring in some items) − 1) × 25).

When converting the basic Likert scale to a 100-point scale, it should be assumed that: 1 = 0, 2 = 25, 3 = 50, 4 = 75, 5 = 100, where 0 is the worst possible level and 100 is the best possible level of the scale. By analysing the above, scores are calculated for two parameters (the teamwork and safety climate) on a scale from 0 to 100 points. Positive answers are those that start with 75 points.

There is also an open-ended question at the end of the questionnaire, in which participants can express their views on how to improve patient safety in a given facility and make other remarks (also regarding the comprehensibility of the statements).

### Data collection

A cross-sectional survey was conducted among the medical and auxiliary personnel of long-term care facilities all over Poland. The study was conducted from July 1, 2021, to May 15, 2022. A stratified random (proportional) selection of facilities for the study was carried out after drawing up a list of all facilities in the country, broken down by voivodeships. Then, one by one, according to the prepared list, the centres were asked for permission from the director/manager to conduct the study. After obtaining permission, the questionnaires were delivered to the facilities by post and then distributed by administrative personnel.

### Statistical analysis

The statistical application SPSS IMB version 26 was used to prepare the analyses. The factor analysis was performed—varimax rotation with Kaiser normalization.

The data set was analysed in terms of adequacy to the factor analysis model through a preliminary analysis of the correlation of variables and the methods provided in literature on the subject: the Kaiser-Mayer-Olkin (KMO) test and the Bartlett test.

The reliability of the factors was tested using Cronbach’s alpha values, with a minimum criterion of 0.70. Corrected item total correlation (CITC) was also calculated.

In addition, the Spearman’s correlation coefficient was used to measure the correlation between the areas of the questionnaire and individual variables related to facility parameters and the respondents.

### Ethical considerations

The study was conducted among employees of long-term care facilities in Poland, observing the principles of ethics. Participants received written information that they could read before the study. It presented the objective of the study and indicated that the answers are anonymous and will only be used for scientific purposes. We confirm that the work and conducted research are in accordance with the Declaration of Helsinki. Informed consent was obtained from all subjects involved in the study. All methods were carried out in accordance with relevant guidelines and regulations.

This study was supported and approved by the Medical University of Warsaw. Permission for the study was obtained from all managers of long-term care facilities where the study was conducted. Due to the fact that the study was not conducted among patients and is not a medical experiment, in accordance with Polish law, the consent of the bioethics committee was not required.

## Results

### Sample

Respondents (n = 558) working in 26 different long-term care facilities participated in the study. The majority of the study sample was made up of nursing personnel (62.7%). Medical caregivers constituted 16.5% of the respondents, physiotherapists 7%, and doctors 6.6% of all respondents. Other people who completed the questionnaire (7.2% of all respondents) were occupational therapists, speech therapists, psychologists and pharmacists (Table 1).

**Table 1:** The position and seniority of respondents.

Variable	N	%
Position		
Nurse	350	62.7%
Medical caregiver	92	16.5%
Physiotherapist	39	7%
Doctor	37	6.6%
Other	40	7.2%
Seniority		
Less than 6 months	24	4.3%
6–11 months	27	4.8%
1–2 years	70	12.6%
3–7 years	130	23.3%
8–12 years	75	13.4%
13–20 years	89	16.0%
21 years and more	125	22.4%

### Psychometric results

The KMO measure suggests that factor analysis of the collected data is the right way to proceed. It shows a fairly high level (> 0.8) indicating that correlations between pairs of variables can be explained by other variables. The value of the KMO statistic is in the range < 0, 1 > . A value close to 1 proves that the internal structure of the tool, identified by factor analysis, is clear and reliable. The result of the Bartlett test suggests that we can reject the null hypothesis that the correlation matrix is an identity matrix (chi2 = 3791; p < 0.01) and accept the alternative hypothesis that there are true relationships between the statements, thus the factor analysis could be performed.

Extracting significant number of main factors had been performed on the basis of so-called internal value criterion, where factor are only significant where internal value are above 1. Based on this criterion, also known as Kaiser’s criterion, four significant main factors were identified. Those factors corresponded with 50% observed variability, where first factor after rotation corresponded with 15.3%, second factor—13.7%, third 11.9%, and fourth 8.9% of the observed variability in the source dataset (Table 2).

**Table 2:** Eigenvalues of factors and percentage of explained variance.

Component	Initial eigenvalues			Extraction sums of squared loadings			Rotation sums of squared loadings		
	Total	% of variance	Cumulative %	Total	% of Variance	Cumulative %	Total	% of variance	Cumulative %
1	8255	30,573	30,573	8255	30,573	30,573	4125	15,277	15,277
2	2037	7546	38,119	2037	7546	38,119	3712	13,748	29,025
3	1728	6399	44,518	1728	6399	44,518	3220	11,925	40,949
4	1435	5313	49,831	1435	5313	49,831	2398	8882	49,831

The Varimax orthogonal rotation analysis results showed that each identified factor has appropriately strong item loading. Factorial loading are all within 0.437–0.798 range, thus each question is strong enough to remain in the questionnaire (Table 3).

**Table 3:** Rotated component matrix.

Item	Component			
	1	2	3	4
The physicians and nurses here work together as a well-coordinated team	0.718			
I have the support I need from other personnel to care for patients	0.644			
I am satisfied with the quality of collaboration that I experience with staff physicians in this clinical area	0.626		0.366	
The culture in this clinical area makes it easy to learn from the errors of others	0.560			
Briefings are common in this clinical area	0.549			0.356
I am encouraged by my colleagues to report any patient safety concerns I may have	0.538	0.303		
It is easy for personnel here to ask questions when there is something that they do not understand	0.537	0.308		
I am satisfied with the quality of collaboration that I experience with nurses in this clinical area	0.533		0.367	
Disagreements in this clinical area are resolved appropriately (i.e., not who is right, but what is best for the patient)	0.464	0.317	0.374	
Leadership is driving us to be a safety-centered institution		0.798		
My suggestions about safety would be acted upon if I expressed them to the management		0.731		
This institution is doing more for patient safety now than it did 1 year ago		0.673		
I receive appropriate feedback about my performance	0.402	0.603		
Medical errors are handled appropriately here	0.374	0.530		
I would feel safe being treated here as a patient		0.524	0.428	
Decision-making in this clinical area utilizes input from relevant personnel		0.484		
In this clinical area, it is difficult to discuss errors			0.775	
I am frequently unable to express disagreement with the attendings/staff physicians here			0.774	
Management does not knowingly compromise patient safety			0.755	
In this clinical area, it is difficult to speak up if I perceive a problem with patient care			0.578	
Personnel frequently disregard rules or guidelines (e.g., handwashing, treatment protocols/clinical pathways, sterile field, etc.) that are established for this clinical area			0.468	
Briefing personnel before start of a shift (i.e., to plan for possible contingencies) is important for patient safety				0.605
Important issues are well communicated at shift changes	0.477			0.579
Nurse input is well received in this clinical area				0.574
I know the proper channels to direct questions regarding patient safety in this clinical area	0.321	0.327		0.526
I know the first and last names of all the personnel I worked with during my last shift				0.504
The levels of staffing in this clinical area are sufficient to handle the number of patients		0.359		0.437

The content interpretation of the factors determined that first factor regards cooperation as part of work. The second factor regards clearly to patient safety provided by the hospital from both management and directly caring for patients personnel perspectives. The third factor is made of questions regarding the ease and ability to speak up about ensuring patient safety. The last factor, fourth, are questions related to patient safety centred work organisation in the facility.

The statements on the teamwork climate scale correlate with the scale from 0.308 to 0.621, so these are low to moderately strong correlations. The reliability of the scale is high (alpha = 0.834). The safety climate scale also shows high reliability, where the questions within the scale have moderately high to high correlation to the scale (0.475–0.681). The reliability of the scale ability to speak up and following the rules is also high (alpha = 0.719). The questions correlate with the scale within 0.335–0.558 range. The work organisation is a scale with unsatisfactory reliability (alpha = 0.577). The question with significant influence on the reliability of the scale (‘The levels of staffing in this clinical area are sufficient to handle the number of patients’)—with the question removed, the reliability of the scale would improve to satisfactory 0.704 (Table 4). Due to high diagnostic value of this question for the nurses’ work market in Poland and challenges of maintaining adequate numbers of medical personnel this question has been left in the questionnaire for further consideration.

**Table 4:** The results of Cronbach’s alpha and CITC.

	Cronbach’s alpha total	Corrected item-total correlation	Cronbach’s alpha if item deleted
Teamwork climate	0.834		
The physicians and nurses here work together as a well-coordinated team		0.673	0.801
Disagreements in this clinical area are resolved appropriately (i.e., not who is right, but what is best for the patient)		0.575	0.813
It is easy for personnel here to ask questions when there is something that they do not understand		0.587	0.812
I have the support I need from other personnel to care for patients		0.577	0.814
Briefings are common in this clinical area		0.457	0.827
I am satisfied with the quality of collaboration that I experience with staff physicians in this clinical area		0.590	0.811
I am satisfied with the quality of collaboration that I experience with nurses in this clinical area		0.577	0.813
I am encouraged by my colleagues to report any patient safety concerns I may have		0.495	0.822
The culture in this clinical area makes it easy to learn from the errors of others		0.379	0.835
Safety climate	0.842		
Decision-making in this clinical area utilizes input from relevant personnel		0.475	0.839
I would feel safe being treated here as a patient		0.570	0.824
I receive appropriate feedback about my performance		0.644	0.813
Medical errors are handled appropriately here		0.575	0.824
This institution is doing more for patient safety now than it did 1 year ago		0.525	0.832
Leadership is driving us to be a safety-centered institution		0.722	0.801
My suggestions about safety would be acted upon if I expressed them to the management		0.681	0.807
Ability to speak up and following the rules	0.719		
In this clinical area, it is difficult to speak up if I perceive a problem with patient care		0.422	0.694
I am frequently unable to express disagreement with the attendings/staff physicians here		0.523	0.653
Personnel frequently disregard rules or guidelines (e.g., handwashing, treatment protocols/clinical pathways, sterile field, etc.) that are established for this clinical area		0.335	0.724
In this clinical area, it is difficult to discuss errors		0.558	0.637
Management does not knowingly compromise patient safety		0.555	0.639
Work organisation	0.577		
Nurse input is well received in this clinical area		0.422	0.484
Important issues are well communicated at shift changes		0.412	0.499
Briefing personnel before start of a shift (i.e., to plan for possible contingencies) is important for patient safety		0.448	0.495
I know the first and last names of all the personnel I worked with during my last shift		0.281	0.550
The levels of staffing in this clinical area are sufficient to handle the number of patients		0.122	0.704
I know the proper channels to direct questions regarding patient safety in this clinical area		0.470	0.473

### Additional analysis

For the newly created scale descriptive statistics have been provided (Table 5). Lowest scores had been noted in the questions regarding the ease of the personnel to speak up about patient safety and following the rules. Highest mean values were noted in regards to work organisation. The teamwork climate and safety climate were scored similarly. It is worth noting, that these are also aspects where lowest possible scores (1 point) were not found, while in the ability to speak up and following rules and work organisation such scores had been given.

**Table 5:** Descriptive statistics of the TSCS questionnaire scales.

Component	Mean	Median	Std. deviation	Minimum	Maximum
Teamwork climate	4.13	4.22	0.65	1.67	5.00
Safety climate	4.11	4.20	0.69	1.71	5.00
Ability to speak up and following the rules	3.63	3.80	0.88	1.00	5.00
Work organisation	4.24	4.33	0.62	1.00	5.00

The analysis of correlations revealed a number of significant correlations between the Teamwork climate, Safety climate, Ability to speak up and following the rules, Work organisation and individual variables corresponding to the parameters of long-term care facilities and the respondents themselves (Table 6).

**Table 6:** Spearman correlation between the areas of the questionnaire and individual variables concerning facility parameters and respondents.

		1	2	3	4	5	6	7	8	9
1	Teamwork climate	1								
2	Safety climate	0.745**	1							
3	Ability to speak up and following the rules	0.515**	0.442**	1						
4	Work organisation	0.636**	0.540**	0.429**	1					
5	Number of patients in the facility (at the time of the study)	−0.037	−0.052	−0.034	0.032	1				
6	Number of employed nurses	−0.075	−0.053	−0.038	0.077	0.594**	1			
7	Nurses specializing in long-term geriatrics	−0.154**	−0.072	−0.196**	−0.062	−0.214**	0.004	1		
8	Number of employed doctors	−0.151**	−0.127**	0.023	−0.038	0.439**	0.323**	−0.287**	1	
9	Number of employed medical caregivers	0.155**	0.077	0.158**	0.118**	0.480**	−0.015	−0.329**	0.281**	1
10	Seniority	0.032	0.042	−0.129**	0.018	−0.013	0.004	−0.001	−0.02	−0.175**

**Correlation is significant at the 0.01 level (2-tailed).

The teamwork climate strongly and positively correlates with safety climate (rho = 0.745), moderately strongly with ability to speak up and following the rules (rho = 0.515) and work organisation (rho = 0.636). There are also negative and very weak correlations between teamwork climate and number of nurses with specialisation in long-term geriatric care (rho = −0.154) and number of employed doctors (rho = −0.151). In contrast, the high scores of the teamwork climate correlate with higher number of employed medical caregivers (rho = 0.155).

The higher the respondents score the safety climate, the higher they score their ease of speaking up about safety at their facility and vice versa (rho = 0.442). High values on the safety climate scale are accompanied by high scores on work organisation scale (rho = 0.540). A very weak negative correlation safety climate and number of employed doctors had been observed (rho = −0.127).

The ability to speak up moderately strongly positively correlates with work organisation score (rho = 0.429). The number of employed medical caregivers positively correlates with capability to speak up (rho = 0.158). High values on work organisation scale is accompanied by high number of employed medical caregivers (rho = 0.118).

Table 7 summarizes the results for individual statements of the questionnaire along with the values of the median, mean and standard deviation. The percentage of missing answers or those that did not apply to the respondents was also presented, and they averaged 2.9% in the entire sample (results oscillated between 0.4% and 8.4%).

**Table 7:** Descriptive statistics for individual TSCS statements.

Statement	N	Missing/NA	Median	Mean	SD
		N (%)	Scale 1–5		
Nurse input is well received in this clinical area	519	39 (7.0)	5	4.50	0.89
In this clinical area, it is difficult to speak up if I perceive a problem with patient care*	546	12 (2.2)	2	2.23	1.33
Decision-making in this clinical area utilizes input from relevant personnel	555	3 (0.5)	4	4.22	1.01
The physicians and nurses here work together as a well-coordinated team	546	12 (2.2)	4	4.10	1.03
Disagreements in this clinical area are resolved appropriately (i.e., not who is right, but what is best for the patient)	553	5 (0.9)	4	3.91	1.08
I am frequently unable to express disagreement with the attendings/staff physicians here*	511	47 (8.4)	2	2.66	1.26
It is easy for personnel here to ask questions when there is something that they do not understand	550	8 (1.4)	4	4.23	0.91
I have the support I need from other personnel to care for patients	544	14 (2.5)	5	4.33	0.86
I know the first and last names of all the personnel I worked with during my last shift	547	11 (2.0)	5	4.68	0.72
Important issues are well communicated at shift changes	529	29 (5.2)	5	4.43	0.77
Briefing personnel before the start of a shift (i.e., to plan for possible contingencies) is important for patient safety	544	14 (2.5)	5	4.63	0.72
Briefings are common in this clinical area	522	36 (6.5)	5	4.31	1.07
I am satisfied with the quality of collaboration that I experience with staff physicians in this clinical area	532	26 (4.7)	4	4.13	0.98
I am satisfied with the quality of collaboration that I experience with nurses in this clinical area	553	5 (0.9)	5	4.31	0.90
The levels of staffing in this clinical area are sufficient to handle the number of patients	550	8 (1.4)	2	2.95	1.36
I would feel safe being treated here as a patient	545	13 (2.3)	4	4.07	0.97
I am encouraged by my colleagues to report any patient safety concerns I may have	551	7 (1.3)	4	4.13	0.94
Personnel frequently disregard rules or guidelines (e.g., handwashing, treatment protocols/clinical pathways, sterile field, etc.) that are established for this clinical area*	551	7 (1.3)	2	2.07	1.21
The culture in this clinical area makes it easy to learn from the errors of others	548	10 (1.8)	4	3.70	1.04
I receive appropriate feedback about my performance	545	13 (2.3)	4	4.03	1.01
Medical errors are handled appropriately here	534	24 (4.3)	4	4.25	0.81
I know the proper channels to direct questions regarding patient safety in this clinical area	542	16 (2.9)	5	4.48	0.75
In this clinical area, it is difficult to discuss errors*	545	13 (2.3)	2	2.51	1.31
Management does not knowingly compromise patient safety	515	43 (7.7)	4	3.62	1.28
This institution is doing more for patient safety now than it did 1 year ago	529	29 (5.2)	4	3.94	0.97
Leadership is driving us to be a safety-centered institution	556	2 (0.4)	4	4.22	0.90
My suggestions about safety would be acted upon if I expressed them to management	545	13 (2.4)	4	4.05	0.95

*Reverse scored item.

## Discussion

The TSCS questionnaire had been utilised in different researches from various healthcare perspectives^30–37^. It has been used, among other, to assess the difference made by implementing in hospitals changes directed at improving patient safety, in areas such as leadership, rounds, implementation of various communication techniques, teamwork building training (testing before and after implementation of the changes)^30–33^, including perinatal and paediatric wards^35–37^. Each cited research had been conducted in an English speaking country (US, United Kingdom, Australia) using the English version of the questionnaire. The authors have not found a study utilising the TSCS questionnaire in any other than English language.

One study had been found where factor structure, reliability, and usability of the English version of TSCS questionnaire had been analysed. That study had been conducted in healthcare facilities in the United Kingdom^21^. The authors had not found any study where TSCS questionnaire had been utilised in long-term care facility.

The translated version of the TSCS questionnaire can be used not only in long-term care facilities, but also in various healthcare facilities including hospitals. The usability aspect of the questionnaire still needs further research in facilities other than providing long-term care.

The results obtained in this study can be indirectly compared with the results of studies conducted in long-term care facilities that used other research tools (mainly the SAQ). In a study conducted in the Netherlands using the SAQ, Cronbach’s alpha was 0.73 for the teamwork climate and 0.76 for the safety climate^27^. In another study, also conducted in long-term care facilities in Norway, the questionnaire was translated, but not the original version, but the version intended for the analysis of patient safety in the outpatient clinic, i.e., SAQ-A. The results of Cronbach’s alpha coefficient in this case were 0.655 for the teamwork climate and 0.738 for the safety climate^24,25^. The above factors may be affected by a different structure, a different number of statements in the scales, and probably the method of linguistic adaptation of the questionnaire.

The correlation analysis showed several correlations. First of all, as expected, the teamwork climate correlates strongly and positively with the safety climate. The above correlation is also confirmed in other publications on patient safety in healthcare facilities, using various tools^27,38–42^. Such a result may also constitute a basis for supposing that improving the teamwork climate may positively affect the safety climate. The more so that there are scientific reports indicating that the number of medical errors, and thus also adverse events, depends on the degree of development of communication skills and teamwork^43–45^.

As a result of the analysis, it was shown that the smaller the number of doctors and nurses with a specialization, the better the teamwork climate. This may be related to the characteristics of the statements in this part of the questionnaire. Smaller, more cohesive teams can work better together and know each other better. In larger facilities that care for more patients, where more personnel is needed, there is a potential for conflicts and poor communication. Therefore, it is important to improve cooperation in therapeutic teams. There are many communication techniques that can help healthcare professionals communicate with other healthcare professionals. One of them is the SBAR (Situation, Background, Assessment, Recommendation) scheme^12,46–48^. Its essence is to present information about the situation and the patient's condition in the most concise and specific way possible, as well as to recommend a course of action. The key is to indicate which patient the message concerns (characteristic features that will allow the identification of the patient), his/her ailments (description of symptoms and a brief presentation of the disease history), functional status (determination of objective parameters, e.g. saturation, blood pressure, rhythm heart, as well as the patient's subjective feelings that he communicates) as well as the actions taken (e.g. names of the drugs given) and further recommendations (suggesting what the problem may be about with a simultaneous request for consultation/confirmation of the course of action). The technique is extremely useful when it is necessary to undertake external consultations related to newly admitted patients, but it is also useful in consultations within well-coordinated therapeutic teams. Another scheme is I-PASS (Illnes severity, Patient summary, Action items, Situation, Synthesis)^49–51^. The strategy can be used during medical staff briefings or medical rounds. The first element is to provide brief information about the patient's condition (indicating whether the patient's condition is unstable or stable). Then, describe the patient's situation, developing information about the clinical situation and indicate the necessary list of actions that should be performed in the near future. The effect of the actions taken is also analyzed (visualization of the patient's condition after the intervention). At the very end, the recipient of the message should summarize the information he or she has heard (paraphrase) and, if he or she does not understand individual elements of the message, should ask questions. Paraphrasing the sender's statement by the recipient should be widely used and constitute a key element of the communication scheme in health care. In addition to paraphrase, it is also possible to use a strategy in which the listener repeats the information obtained from the sender of the message unchanged. The technique can be very useful when issuing medical orders in crisis situations.

One of the important elements influencing the level of patient safety culture is the willingness to report adverse events and learn from errors. Although in the question "The culture in this clinical area makes it easy to learn from the errors of others" most people answered positively, improvement in this area is possible. In order to increase the knowledge of medical staff regarding adverse events, it is possible to introduce appropriate educational techniques aimed at reducing the number of medical errors. In each facility, it would be reasonable to implement an appropriate training model devoted to the importance of reporting incidents and methods of preventing them. Implementing methods described in the literature, i.e. meetings during which cases of adverse events are discussed (morbidity and mortality meetings) held e.g. monthly among the facility's medical staff, or talks with interactive elements, may contribute to improving patient safety^52^. It would therefore be possible to introduce new elements within existing training courses related to further reduction of the risk of adverse events.

When discussing research results, the potential impact of the Covid-19 pandemic on the results should also be taken into account. First of all, the devastation caused by the SARS-CoV-2 virus in nursing homes made medical staff aware of the high risk of care provided in this type of facilities. Secondly, the epidemiological situation has led to closer cooperation between various entities influencing the education of medical staff and introducing changes aimed at increasing patient safety^53^. A thorough analysis of the impact of the Covid-19 pandemic on the patient safety culture in long-term care facilities in Poland would only be possible by directly comparing the situation in specific facilities before and during the outbreak of the pandemic (using questionnaires such as the TSCS). However, reliable data from Polish facilities before the outbreak of the pandemic are unavailable. At the same time, when analyzing this issue, it is worth emphasizing that one of the issues included in the TSCS questionnaire was "This institution is doing more for patient safety now than it did 1 year ago". The answers to the above question indicate that most employees are of the opinion that more activities are being undertaken in the facility to improve safety compared to a year ago. Therefore, further research should be conducted in long-term care facilities in Poland in the future.

The limitation of this study was undoubtedly the method of delivering the questionnaires to the institutions. The ongoing Covid-19 pandemic prevented the personal delivery and collection of questionnaires from medical personnel. During the period in which the study was conducted, there were significant restrictions in access to health care facilities, in particular long-term care facilities where people at risk of infection stay. The imposed restrictions prevented people who did not work there from entering the facilities. Therefore, the questionnaires were distributed on the premises by managers or persons appointed by them, which was potentially a factor increasing the level of fear of medical personnel before completing the questionnaires truthfully. This could have resulted in overestimating the results obtained in individual statements. Moreover, it is likely that only facilities that place a high value on patient safety chose to participate in the study. A large number of facilities refused to participate in the study (28 facilities) or did not send the questionnaires back to the author of the study (34 facilities), which may indicate an unfavourable situation related to patient safety in these facilities. The limitation of the study was lack of conducting a repeated testing, thus the authors were unable to comprehensively assess the reliability of the questionnaire.

## Conclusions

Based on our study, it can be concluded that the Polish version of the TSCS may be an useful tool for measuring the climate of teamwork and safety in long-term care facilities. The results obtained from filled questionnaire can be valuable source of information on the patient safety culture for facility management. The authors recommend further studies with Polish translation of the TCSC questionnaire on a larger group of medical personnel.

### Supplementary Information

Supplementary Information.

## Data Availability

The datasets generated during and/or analysed during the current study are available from the corresponding author on reasonable request.
