# Supplementary material for: Teamwork and safety climate in Polish long-term care facilities: questionnaire reliability and usability
Source: Sci Rep. 2023 Nov 30;13:21115. doi: 10.1038/s41598-023-48415-8 (PMC10689842; doi:10.1038/s41598-023-48415-8)
Supplement: Supplementary file 1 — Supplementary Information. [file 41598_2023_48415_MOESM1_ESM.docx]

| **W celu prawidłowego zaznaczenia wybranej odpowiedzi zaznacz krzyżykiem prostokąt z literą - bez wychodzenia za jego obwód.** | | | | | | | | | | | | | | | | | | | |
| --- | --- | --- | --- | --- | --- | --- | --- | --- | --- | --- | --- | --- | --- | --- | --- | --- | --- | --- | --- |
| **Proszę odpowiedzieć na poniższe sentencje w odniesieniu do miejsca pracy w którym otrzymali Państwo ankietę.** | | | | | | | |  |  | **X** | **To pytanie nie ma zastosowania w moim przypadku** | | | | | | | | |
|  |  |  |  |  |  |  |  |  | **E** |  |  |  |  | **Zgadzam się zdecydowanie** | | | | |  |
|  |  |  |  |  |  |  |  | **D** |  |  |  |  |  | **Raczej Zgadzam się** | | | |  |  |
|  |  |  |  |  |  |  | **C** |  |  |  | **Ani zgadzam Ani nie zgadzam się** | | | | | |  |  |  |
|  |  |  |  |  |  | **B** |  |  |  |  |  | **Raczej Nie zgadzam się** | | | |  |  |  |  |
|  |  |  |  |  | **A** |  |  |  | **Nie zgadzam się zdecydowanie** | | | | | |  |  |  |  |  |
|  |  |  |  |  |  |  |  |  |  |  |  |  |  |  |  |  |  |  |  |
|  |  |  |  |  |  |  |  |  |  |  |  |  |  |  |  |  |  |  |  |
| 1. | Wprowadzenie pielęgniarki w pracę w zakładzie jest u nas ogólnie przyjętym standardem | | | | | | | | | | | | | A  B  C  D  E  X |  |  | A |  |  |
| 2. | W moim miejscu pracy trudno jest mi „mówić głośno” o napotkanych przeze mnie problemach w opiece nad pacjentem | | | | | | | | | | | | | A  B  C  D  E  X |  |  |  |  |  |
| 3. | W moim miejscu pracy wszelkie decyzje są podejmowane przy współudziale właściwych do tego osób | | | | | | | | | | | | | A  B  C  D  E  X |  |  |  |  |  |
| 4. | W moim zakładzie lekarze i pielęgniarki współpracują jak zgrany zespół | | | | | | | | | | | | | A  B  C  D  E  X |  |  |  |  |  |
| 5. | W moim zakładzie kwestie sporne są rozstrzygane we właściwy sposób,  tzn. nieważne „KTO” reprezentuje daną rację, ale czy ta racja jest słuszna | | | | | | | | | | | | | A  B  C  D  E  X |  |  |  |  |  |
| 6. | W moim zakładzie często jest tak, że nie jestem w stanie wyrazić sprzeciwu wobec decyzji personelu lekarskiego | | | | | | | | | | | | | A  B  C  D  E  X |  |  |  |  |  |
| 7. | Osoby pracujące w moim zakładzie odczuwają łatwość w zadawaniu pytań jeśli coś jest dla nich niezrozumiałe | | | | | | | | | | | | | A  B  C  D  E  X |  |  |  |  |  |
| 8. | Inni pracownicy zakładu udzielają mi wsparcia, które jest mi potrzebne w opiece nad pacjentami | | | | | | | | | | | | | A  B  C  D  E  X |  |  |  |  |  |
| 9. | Znam imiona i nazwiska wszystkich osób z którymi ostatnio pracował-am/em na zmianie | | | | | | | | | | | | | A  B  C  D  E  X |  |  |  |  |  |
| 10. | Podczas zdawania dyżuru/raportu, istotne kwestie są dobrze zaakcentowane przez osoby schodzące ze zmiany | | | | | | | | | | | | | A  B  C  D  E  X |  |  |  |  |  |
| 11. | Odprawa personelu przed rozpoczęciem zmiany (tj. zasygnalizowanie możliwych komplikacji) ma istotne znaczenie dla bezpieczeństwa pacjenta | | | | | | | | | | | | | A  B  C  D  E  X |  |  |  |  |  |
| 12. | Odprawa personelu w moim zakładzie to powszechne zjawisko | | | | | | | | | | | | | A  B  C  D  E  X |  |  |  |  |  |
| 13. | Jestem usatysfakcjonowan-a/y z jakości współpracy, której doświadczam w trakcie pracy z l e k a r z a m i z mojego zakładu | | | | | | | | | | | | | A  B  C  D  E  X |  |  |  |  |  |
| 14. | Jestem usatysfakcjonowan-a/y z jakości współpracy, której doświadczam w trakcie pracy z p i e l ę g n i a r k a m i z mojego zakładu | | | | | | | | | | | | | A  B  C  D  E  X |  |  |  |  |  |
| 15. | Liczba zatrudnionego personelu w moim zakładzie jest adekwatna do liczby pacjentów | | | | | | | | | | | | | A  B  C  D  E  X |  |  |  |  |  |
| 16. | Będąc pacjentem mojego zakładu miał-abym/bym poczucie bezpieczeństwa odnośnie otrzymywanego leczenia/opieki | | | | | | | | | | | | | A  B  C  D  E  X |  |  |  |  |  |
| 17. | Jestem zachęcan-a/y przez moich współpracowników do zgłaszania jakichkolwiek obaw dotyczących bezpieczeństwa pacjenta | | | | | | | | | | | | | A  B  C  D  E  X |  |  |  |  |  |
| 18. | Pracownicy tego zakładu często lekceważą/nie przestrzegają ustanowionych zasad i wytycznych postępowania (np. mycia rąk, ścieżek klinicznych, procedur, antyseptyki) | | | | | | | | | | | | | A  B  C  D  E  X |  |  |  |  |  |
| 19. | Kultura panująca w moim zakładzie sprawia, że łatwo jest uczyć się na błędach innych członków zespołu | | | | | | | | | | | | | A  B  C  D  E  X |  |  |  |  |  |
| 20. | Otrzymuję konstruktywną/merytoryczną informację zwrotną odnośnie wykonywanej przeze mnie pracy | | | | | | | | | | | | | A  B  C  D  E  X |  |  |  |  |  |
| 21. | W moim zakładzie radzimy sobie z błędami medycznymi we właściwy sposób | | | | | | | | | | | | | A  B  C  D  E  X |  |  |  |  |  |
| 22. | Wiem w jaki sposób i do kogo, kierować pytania dotyczące bezpieczeństwa pacjenta w moim miejscu pracy | | | | | | | | | | | | | A  B  C  D  E  X |  |  |  |  |  |
| 23. | W moim miejscu pracy trudno jest dyskutować/wymieniać poglądy na temat popełnianych błędów | | | | | | | | | | | | | A  B  C  D  E  X |  |  |  |  |  |
| 24. | Kierownictwo zakładu nie przyczynia się świadomie do pogorszenia bezpieczeństwa pacjenta | | | | | | | | | | | | | A  B  C  D  E  X |  |  |  |  |  |
| 25. | Moja instytucja (zakład) obecnie robi więcej na rzecz bezpieczeństwa pacjenta niż robiła rok temu | | | | | | | | | | | | | A  B  C  D  E  X |  |  |  |  |  |
| 26. | Kierownictwo zakładu prowadzi nas w kierunku instytucji, która stawia bezpieczeństwo pacjenta w centrum uwagi | | | | | | | | | | | | | A  B  C  D  E  X |  |  |  |  |  |
| 27. | Moje sugestie dotyczące bezpieczeństwa pacjenta mogłyby być pozytywnie rozpatrzone gdybym tylko przedstawi-ł/ła je osobom zarządzającym | | | | | | | | | | | | | A  B  C  D  E  X |  |  |  |  |  |

|  | | | | | | | | | | | | | | | | | | | |
| --- | --- | --- | --- | --- | --- | --- | --- | --- | --- | --- | --- | --- | --- | --- | --- | --- | --- | --- | --- |
|  |  |  |  |  |  |  |  |  |  |  |  |  |  |  |  |  |  |  |  |
|  |  |  |  |  |  |  |  |  |  |  |  |  |  |  |  |  |  |  |  |
| **Określ proszę swoją pozycję zawodową w zakładzie, w którym wręczono Ci ankietę**  **(wielokrotny wybór w przypadku zawodu pielęgniarki i położnej):** | | | | | | | | | | | | | | | | | | | |
|  |  |  |  |  |  |  |  |  |  |  |  |  |  |  |  |  |  |  |  |
| 🞏 Lekarz ze specjalizacją | | | | | |  | 🞏 Pielęgniarka  – średnie medyczne | | | | |  | 🞏 Położna  – średnie medyczne | | | | | |  |
|  |  |  |  |  |  |  |  |  |  |  |  |  |  |  |  |  |  |  |  |
| 🞏 Lekarz bez specjalizacji | | | | | |  | 🞏 Pielęgniarka  – wyższe (lic./mgr pielęgniarstwa) | | | | | | 🞏 Położna  – wyższe (lic./mgr położnictwa) | | | | | |  |
|  |  |  |  |  |  |  |  |  |  |  |  |  |  |  |  |  |  |  |  |
| 🞏 Lekarz rezydent | | | | | |  | 🞏 Pielęgniarka  – ze specjalizacją | | | | |  | 🞏 Położna  – ze specjalizacją | | | | | |  |
|  |  |  |  |  |  |  |  |  |  |  |  |  |  |  |  |  |  |  |  |
| 🞏 Lekarz stażysta | | | | | |  | 🞏 inna, proszę określ …………………………………….…………… | | | | | | | | |  |  |  |  |
|  |  |  |  |  |  |  |  |  |  |  |  |  |  |  |  |  |  |  |  |
| **Określ swój staż pracy w zakładzie, w którym wręczono Ci ankietę:** | | | | | | | | | | | | | | | | | | | |
|  |  |  |  |  |  |  |  |  |  |  |  |  |  |  |  |  |  |  |  |
| 🞏 mniej niż 6 miesięcy | | | | | | 🞏 6-11 miesięcy | | | | | 🞏 1-2 lata | | | | 🞏 3-7 lat | | | |  |
|  |  |  |  |  |  |  |  |  |  |  |  |  |  |  |  |  |  |  |  |
|  |  | 🞏 8-12 lat | | | | |  | 🞏 13-20 lat | | | | |  | 🞏 21 lat i więcej | | | | |  |
|  |  |  |  |  |  |  |  |  |  |  |  |  |  |  |  |  |  |  |  |
| **Wskaż województwo, w którym znajduje się placówka:**  ……………………………………………………………………  **Określ profil placówki:**  …………………………………………………………………… | | | | | | | | | |  | | | | | | | | | |
|  |  |  |  |  |  |  |  |  |  |  |  |  |  |  |  |  |  |  |  |
|  |  |  |  |  |  |  |  |  |  |  |  |  |  |  |  |  |  |  |  |
| Tutaj jest miejsce na Twój komentarz odnośnie bezpieczeństwa pacjenta, błędów medycznych, kultury bezpieczeństwa, organizacji pracy itp., w Twoim zakładzie.  Możesz zostawić również komentarz odnośnie tej ankiety. | | | | | | | | | | | | | | | | | | | |
|  |  |  |  |  |  |  |  |  |  |  |  |  |  |  |  |  |  |  |  |
|  | | | | | | | | | | | | | | | | | | | |
| **Dziękujemy za wypełnienie ankiety**  **Doceniamy Twój czas i udział w badaniu.** | | | | | | | | | | | | | | | | | | | |
